# Prevalence and risk factors for the development of chronic postoperative pain after cataract surgery in the Age-related Eye Disease Study (AREDS)

**DOI:** 10.1016/j.jpain.2025.104790

**Published:** 2025-01-16

**Authors:** Rony R. Sayegh, Susan Vitale, Elvira Agrón, John T. Farrar, Penny A. Asbell, Emily Y. Chew

**Affiliations:** a Cole Eye Institute, Cleveland Clinic, Cleveland, OH, United States; b Division of Epidemiology and Clinical Applications, National Eye Institute, National Institutes of Health, Bethesda, MD, United States; c University of Pennsylvania Perelman School of Medicine, Philadelphia, PA, United States; d University of Memphis, Memphis, TN, United States

**Keywords:** Chronic postoperative pain, Pain, Cataract surgery, Clinical trial, AREDS

## Abstract

Chronic ocular pain impacts quality of life and is often linked to ocular surgery. We assessed the prevalence of chronic postoperative pain (CPOP) after cataract surgery and associated risk factors using a secondary cohort post-hoc analysis of data from the Age-Related Eye Disease Study (AREDS), a multicenter, controlled, randomized clinical trial of antioxidant vitamins and minerals. Ocular pain was determined from item 4 of the National Eye Institute Visual Function Questionnaire (NEI-VFQ-25), administered between 1997 and 2005. We included participants who underwent cataract surgery during the study and reported no or mild ocular pain before first-eye cataract surgery (n=325). Controls (n=283) reported no or mild ocular pain 3 or more months after first-eye cataract surgery; cases (n=42) reported moderate or severe pain 3 or more months after first-eye cataract surgery. Multivariable logistic regression models assessed associations between potential risk factors (age, sex, body mass index, smoking, diabetes, education level, use of anti-inflammatory agents, use of antacids, general health, AREDS treatment group) and CPOP. Of the 325 participants (mean age, 69.7±4.4 years, 59.4 % female); CPOP developed in 42 (13 %; 95 % CI, 9.3 – 16.6 %). The average time between cataract surgery and the post-surgery VFQ was 18.4±11.8 months (range 3.0 – 65.0 months). Multivariable analysis did not reveal any statistically significant associations with odds of developing CPOP after cataract surgery. As such, in this AREDS cohort who underwent cataract surgery, 13% developed CPOP, consistent with previous reports from cataract and refractive surgery. Our post-hoc analyses did not identify any significant risk factors for CPOP.

**Perspective:**

We found a high prevalence of Chronic Postoperative Pain (CPOP) in the AREDS cohort, with 13 % of participants who underwent cataract surgery developing CPOP. Post-hoc analysis did not identify significant risk factors for CPOP. Our study contributes valuable insights into a growing area of interest in pain management within ophthalmology.

## Introduction

Chronic ocular pain is a prevalent condition that significantly impacts quality of life and has substantial implications for global healthcare costs.^1^ A variety of insults, such as surgical injury and ocular surface inflammation, can change the morphology and function of nociceptors, leading to chronic ocular surface pain, even in the absence of ocular surface abnormalities.^2^

Cataract surgery is one of the most commonly performed surgical procedures and much progress has been made towards making it one of the safest. One of the less discussed but significant complications of cataract surgery is chronic postoperative pain (CPOP), which can masquerade as dry eye.^3^

The definition of CPOP was standardized in 2019 after its inclusion in the new International Classification of Diseases, Eleventh Revision (ICD-11).^4^ It is defined as pain that develops or increases in intensity after a surgical procedure, and that persists beyond the healing process, i.e., more than 3 months after the triggering event. It localizes either to the surgical site or is projected onto the innervation area of a nerve in this area, and can often show characteristics of neuropathic pain. Other causes of pain, such as pre-existing pain conditions, infection, and malignancy, are excluded. The prevalence of CPOP after cataract surgery, understanding of its causative factors and associated risks, and tools for accurate diagnosis and treatment remain unelucidated.

To our knowledge, no studies to date have looked specifically at CPOP after cataract surgery. Persistent dry eye symptoms, believed to be a manifestation of CPOP, were found to be present in a significant number of patients after cataract surgery.^3,5^ Discrepancies in persistent dry eye signs and symptoms in bilateral pseudophakic patients further support the belief that the symptoms experienced by these patients are not always related to dry eye, and are rather a manifestation of CPOP.^6^ However, a knowledge gap remains regarding chronic pain experienced by patients after cataract surgery, including its prevalence and associated risk factors.

The Age-Related Eye Disease Study (AREDS) was a multicenter, randomized-controlled clinical trial aimed at assessing the impact of micronutrient supplementation on the development and advancement of cataract and age-related macular degeneration. The primary findings from this trial have been published.^7,8^ This study enrolled participants with a broad spectrum of AMD, ranging from no AMD to unilateral late AMD. Participants eligible for inclusion included those with no lens opacity, lens opacities that allowed sufficient quality of color fundus imaging, or aphakia/pseudophakia. While investigating the progression of cataracts and age-related macular degeneration in this cohort, health-related quality-of-life outcomes were measured using the National Eye Institute Visual Function Questionnaire (NEI-VFQ), which includes information about ocular pain.^9,10^

In this study, we aimed to determine the prevalence of CPOP by studying the AREDS cohort and to assess possible risk factors for CPOP.

## Methods

### Study population

The AREDS was a prospective, multicenter study investigating the clinical course of age-related macular degeneration (AMD) and cataracts, as well as a randomized controlled trial designed to assess the effects of nutritional supplements on AMD and cataract progression.^11^ Briefly, 4757 participants aged 55 to 80 years were recruited between 1992 and 1998 at 11 retinal specialty clinics in the United States. Of these, 128 participants who had bilateral aphakia or pseudophakia participated only in the randomized clinical trial for AMD. The remaining 4629 took part in the cataract trial, and were randomly assigned to placebo, antioxidants, zinc, or the combination of antioxidants and zinc, depending on AMD status.^8^ The primary endpoint for the cataract trial was visual acuity loss and the secondary outcomes included progression of the lens opacities (by the 3 opacity types) and progression to cataract surgery. The AREDS cohort was followed between 1992 and 2005, during which questionnaires, regular eye examinations, and genotyping were performed. Beginning in December 1997, the NEI-VFQ plus appendix was administered to 4119 AREDS participants on at least 2 occasions at least 1 year apart.^8^ No approval by the Institutional Review Board was obtained and subjects were not reconsented for this secondary analysis study as only publicly available deidentified data was used (link in diclosures).

### Eligibility criteria

In our secondary analysis, AREDS participants who had cataract surgery after their baseline AREDS study visit were included. If both eyes had cataract surgery, only the first surgery was used. To be eligible for inclusion, participants had to have the Visual Function Questionnaire (VFQ-25) administrated at least once before, and once after cataract surgery. The postoperative VFQ-25 had to be administered 3 or more months after surgery.

To determine the presence of ocular pain, we used question 4 of the VFQ-25 (see supplement).^12^ The results of the VFQ-25 at the visit closest to (and before) the date of cataract surgery, and the one closest to (and after) 3 months post cataract surgery, were used.

**Fig. 1.**
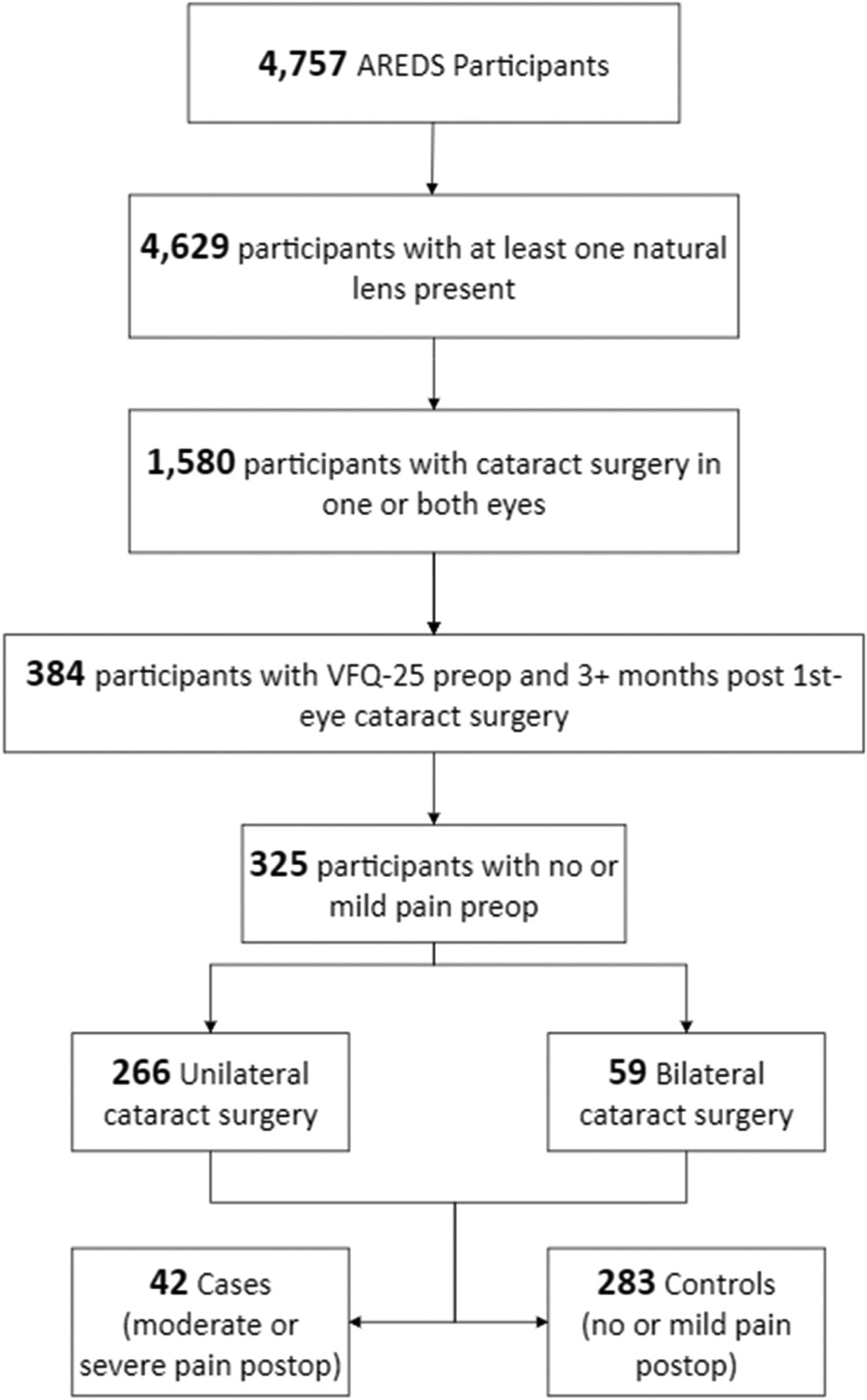
Age-Related Eye Disease Study (AREDS) patient selection schema. (VFQ-25: Visual Function Questionnaire 25).

Cases were defined as individuals with a preoperative score on question 4 of the VFQ-25 of 1 or 2 (none or mild pain) and a postoperative score of 3 or 4 (moderate or severe pain), i.e., those who reported worse pain after cataract surgery. Controls were defined as individuals with a preoperative score of 1 or 2 (none or mild pain) and a postoperative score of 1 or 2 (none or mild pain), i.e., those with no increase in pain score after cataract surgery. (Fig. 1).

### Patient involvement

The results of the AREDS trial were widely disseminated to patients, using informational material and several websites such as the NIH website.

### Statistical analysis

Means and standard deviations were used to describe the baseline characteristics of case and control groups. For risk factor determination, univariable comparison of controls with cases was performed, followed by multivariable logistic regression models to estimate the odds of being a case, relative to a control, while adjusting for demographics, medical history, and AREDS treatment group information (age, sex, BMI, diabetes, smoking, level of formal education, self-reported general health (VFQ question), any use of anti-inflammatories, any use of antacids, and treatment group. Treatment group was categorized in 3 different ways: as 4 levels (placebo, Ax only, Zn only, or Ax+Zn), 2 levels (Zn vs no Zn, i. e., combining both arms containing Zn and comparing with the combined no-Zn arms), or 2 levels (Ax vs no Ax, i.e., combining both arms containing Ax and comparing with the combined no-Ax arms). A full model containing each of the covariates was conducted separately for each of the three treatment group variables. Any variable with a p-value of <0.10 was retained, along with age, in a reduced model. Additionally, after exploring associations among various covariates to identify potential confounders, a model was identified that included age, education level (any college vs no college), diabetes, and treatment group. This reduced model was run for each of the three treatment group variables. All analyses were performed using SAS statistical software (version 9.4; SAS Institute, Cary, NC) and statistical significance was defined as p<0.05.

## Results

325 participants had cataract surgery on their first eye during the cohort’s 5-year follow-up period, had no or mild pain preoperatively, and had the VFQ-25 administered before surgery. The mean age was 69.7±4.4 years, 59.4% were female, and 97.2% were white. The mean time from the 1st VFQ-25 to cataract surgery was 17.02±11.72 months (range, 0–53 months), from cataract surgery to the 2nd VFQ-25 administration 18.46±11.65 months (range 3–65 months), and from the 1st to the 2nd VFQ 38.48±14.02 months (range, 11–80 months). Compared to the 876 participants who had cataract surgery but did not have VFQ data both before and after cataract surgery (i.e., those who were excluded), the only significant differences noted between these groups were for age and BMI, but the differences were quite small and not clinically significant (Supplemental Table 1). Ocular pain 3 or more months after cataract surgery was present in 42 (13%) of the 325; these 42 were designated as cases, and the remaining 283 as controls, for further analyses.

We compared baseline characteristics of cases (n=42) and controls (n=283) (Table 1). Compared with controls, cases were slightly older, more likely to have diabetes, less likely to have any post-high-school education, and more likely to be in one of the treatment arms that included zinc (Zn) (either Zn only or Zn with antioxidant (Ax)).

We used multivariable logistic regression models to estimate the odds of being a case, relative to a control, with a full model containing each of the covariates run separately for each of the three treatment group variables, followed by a reduced model which only included variables with a p-value of <0.10, along with age (Supplemental Table 2).

**Fig. 2.**
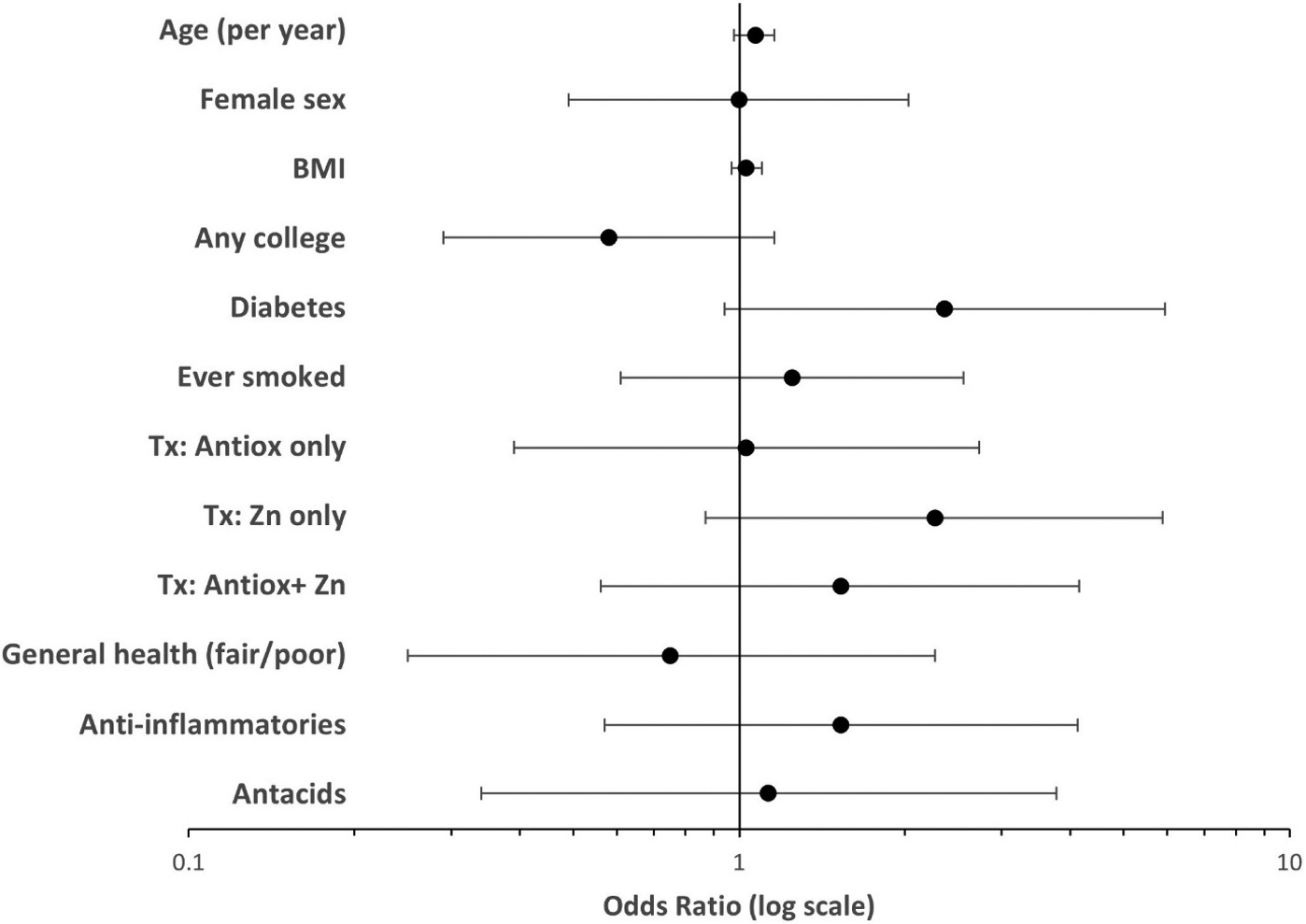
Forest plot of the final model, with treatment group as a 4-level variable, showing diabetes and assignment to the zinc-only treatment group to be borderline significantly associated with increased odds of being a case. (dots represent odds ratios, horizontal lines represent the 95% confidence intervals. BMI: body mass index, Tx: treatment group, Zn: zinc, Antiox: antioxidant).

After exploring associations among various covariates to identify potential confounders, we identified a model that included age, education level (any college vs no college), diabetes, and treatment group, and ran this standard model for each of the three treatment group variables (Table 2 and Fig. 2).

For the final models, with treatment group as a 4-level variable, diabetes (OR 2.27, 95% CI 0.93 – 5.57, p=.07) and assignment to the zinc-only treatment group (OR 2.32, 95% CI, 0.91 – 5.95, p=.06) were borderline significantly associated with increased odds of being a case.

With treatment group as a 2-level variable (any Zn vs no Zn), having any college education was borderline significantly associated with not being a case (OR, 0.56, 95% CI, 0.29 – 1.09, p=.09) and having diabetes (OR, 2.21, 95% CI, 0.91 – 5.38, p=.08) and being in either of the Zn treatment arms (OR, 1.87, 95% CI, 0.96 – 3.64, p=.07) were borderline significantly associated with increased risk of being a case.

With treatment group as a 2-level variable (any Ax vs no Ax), having any college education was borderline significantly associated with not being a case (OR, 0.56, 95% CI, 0.29 – 1.09, p=.09) and increased age (OR=1.07/year, 95% CI, 0.99 – 1.16, p=.09) and having diabetes (OR, 2.25, 95% CI, 0.93 – 5.44, p=.07) were borderline significantly associated with increased risk of being a case.

Variables not found to have a significant association with case status in any of the full models included sex, smoking, BMI, general health (fair or poor), use of anti-inflammatories, and use of antacids.

It is notable that none of the associations were statistically significant at the p=0.05 level, and that if adjustment were made for multiple comparisons (i.e., the multiple ways of categorizing treatment group), none of the associations remained even borderline statistically significant.

## Discussion

We found the prevalence of CPOP after cataract surgery to be 13%, based on a secondary analysis of the AREDS cohort. Prior studies have focused on persistent dry eye symptoms after cataract surgery as a surrogate for ocular pain. Iglesias *et al*. defined persistent post-surgical pain after cataract surgery as “mild or greater dry eye-like symptoms 6 months after surgery” and found that 32% of patients scored 6 or more on the Dry Eye Questionnaire 5 (DEQ5), while 10% reported severe symptoms (DEQ5≥12), similar to our finding.^5^ A similar study by the same group showed frequency of DEQ5≥6 in 34% in their patient population, and of a DEQ5≥12 of 18%, again similar to our study.^3^ These studies were done on a relatively small number of patients, with 86 and 119 participants, respectively, compared to 325 in our study, and the frequencies are in line with other head and face surgeries such as dental implants (8.5–36%).^13^ In larger cohorts, a history of cataract surgery has been found to be a risk factor for having dry eye, including in the British TwinsUK cohort which enrolled 3824 women (OR 1.69 [1.16–2.47] of having a diagnosis of DED by a clinician and currently using artificial tears, p=0.006)^14^ and Lifelines Cohort Study of the Netherlands which had 79,866 voluntary participants (OR 2.60 [2.20–3.07] of having symptomatic dry eye, p<0.001).^15^ While the source of CPOP in these patients remains not fully elucidated, nociceptive, neuropathic, and nociplastic factors are presumed to be involved.^16^ Surgical trauma releases inflammatory mediators like prostaglandins and cytokines, which can sensitize sensory nerves and cause pain. In some cases, this inflammation may persist, leading to plastic changes in the trigeminal ganglion. Perhaps more impactful is the effect of the inevitable nerve injury that occurs when making corneal incisions. Several mechanisms have been postulated to explain CPOP after nerve injury, including ectopic activity in both injured and neighboring uninjured peripheral nociceptive afferents, increased expression of sodium channels, and dysregulation of certain receptor proteins. In addition, central sensitization can occur, involving pro-inflammatory cytokine release, loss of GABAergic interneurons, and coupling between adrenergic sympathetic and nociceptive afferent fibers.^17^

The main limitation of the two previously listed studies is the use of the DEQ-5, a dry eye questionnaire, as a surrogate for CPOP. The DEQ-5 consists of 5 questions graded from 0 (never have it) to 5 (very intense/constantly), with 3 questions pertaining to the frequency of eye discomfort, dry eye feeling, and watery eyes, and 2 questions asking to rate the intensity of the eye discomfort and dry eye feeling. A better measure of ocular pain is the Numeric Rating Scale (NRS), a validated 11-point scale rating the average ocular pain over the prior week. The NRS was used to estimate the prevalence of CPOP after refractive surgery in one study and found that 11% of the 109 patients reported an NRS≥3 at both the 3 and 6-month visits after LASIK or PRK.^18^

The AREDS study used the VFQ-25 questionnaire, which includes a question about the severity of pain or discomfort in or around the eyes, on a scale from 1–5, without a specific time frame.^10^ The VFQ-25 was instituted later in the study protocol (1997), limiting the number of patients who received it both before and after their cataract surgery. Yet our sample size is the largest to date and our study is the first to use a pain question for the CPOP prevalence calculation. It is reassuring that the prevalence of CPOP in our study is consistent with prior studies, confirming the validity of the questionnaire used, and lending further endorsement to our results. It is notable that the prevalence of CPOP remained unchanged in our cohort from the late 1990s compared to more recent publications, despite a significant evolution in the technique and anesthesia used for cataract surgery. This perhaps indicates that surgery and anesthesia-related factors play a lesser role in the development of CPOP after cataract surgery, which is consistent with the mixed evidence linking these factors to chronic post-surgical pain in other parts of the body.^19^

Although we did not find any statistically significant risk factors for the development of CPOP, we did observe non-statistically significant odds ratios of greater magnitude (approximately 2.0) for diabetes, assignment to a treatment arm containing zinc, and having lower level of formal education. The lack of statistical significance may be due in part to the relatively small number of cases, the heterogeneity of the patient population in a retrospective study with less strict inclusion/exclusion criteria, or may simply indicate no correlation; these associations are interesting to explore in future studies designed specifically for CPOP after cataract surgery.

Risk factors for CPOP previously reported in the literature include being a younger adult, less educated, smoker, seeking compensation from their pain, with a history of multiple medical comorbidities, anxiety, depression, pain catastrophizing, preoperative pain which interferes with physical functioning, prior disability, longer duration of surgery and complications, and a higher intensity acute pain after surgery that lasts longer than 5 days.^19^ Specifically for persistent dry eye-like symptoms after cataract surgery, the presence of a non-ocular pain diagnosis and sleep apnea were significantly associated with an increased risk of severe symptoms, but neither remained significantly associated with severe dry eye-like symptoms when considering all other co-variables in the model. Interestingly, several other factors, including depression, PTSD, and the use of an anti-depressant or anxiolytic medication, had an elevated OR but did not reach statistical significance.^5^

A significant strength of this study is its large cohort from the AREDS study, which includes a diverse group of patients with various backgrounds and health conditions, particularly focusing on nutritional supplements. However, the AREDS study was not specifically designed to investigate ocular pain, leading to several limitations. Many risk factors for CPOP and chronic ocular pain that were reported in the literature were not collected in the AREDS study, which focused on vascular risk factors, anti-inflammatory use, and nutritional supplementation, but did not collect information about psychological factors or use of psychiatric medications. Additionally, while question 4 of the VFQ-25 is similar to the gold standard Numeric Rating Scale (NRS), it has not been assessed as an independent pain questionnaire. We elected to use it rather than the pain subscale of the VFQ-25, which includes in addition question 19 “How much does pain or discomfort in or around your eyes, for example, burning, itching, or aching, keep you from doing what you’d like to be doing?” thereby possibly diverting the focus from the intensity of the pain. In our study, the VFQ-25 was administered at 2 single points in time, at the visit before and closest to the surgery, and the visit after and closest to 3 months after surgery. These 2 points were far apart in many cases, and only a subset of patients received the questionnaire as it was introduced later during the study. This limited our patient population and made it difficult to attribute pain specifically to cataract surgery or make specific inferences about the persistent nature of the pain. Finally, the relatively small sample size limited the statistical significance of the various risk factors, especially after assigning subjects to the various supplement arms.

Chronic pain is present in a significant portion of patients after undergoing cataract surgery, one of the most commonly performed surgeries worldwide. There is a pressing need for studies specifically designed for the identification of risk factors, biomarkers, prevention, and treatment strategies, to mitigate the development of chronic pain after cataract surgery. Our exploratory study may aid in hypothesis generation for such future studies.

## Supplementary Material

1

2

3

## Figures and Tables

**Table 1:** Univariable comparison of controls (no increase in pain score after cataract surgery) with cases (increase in pain score after cataract surgery).

Characteristic	Controls (n=283)			Cases (n=42)			Test	p
	Mean	SD	Range	Mean	SD	Range		
**Age (y)**	69.6	4.4	58–79	70.9	4.1	62–78	t-test	0.07
							Wilcoxon	0.09
**BMI**	27.7	5	18.1–58.4	28.8	5.4	20.8–45.6	t-test	0.20
							Wilcoxon	0.23
	**Category**	**No**.	**%**		**No**.	**%**		
**White race**		274	96.8		42	100	Fisher’s*	0.61
**Female sex**		170	60.1		23	54.8	χ^2^	0.51
**Smoking 3-level**	Never	130	45.9		16	38.1	χ^2^	0.57
	Quit	137	48.4		24	57.1		
	Current	16	5.6		2	4.8		
**Smoking 2-level**	Never	130	45.9		16	38.1	χ^2^	0.34
	Ever	153	54.1		26	61.9		
**Diabetes**	Yes	26	9.2		8	19.0	χ^2^	0.05
**Education 3-level**	<=HS°	91	32.3		20	47.6	χ^2^	0.13
	Some college	91	32.3		12	28.6		
	College grad	100	35.5		10	23.8		
**Education 2-level**	<College grad	182	64.5		32	76.2	χ^2^	0.14
	College grad	100	35.5		10	23.8		
**Education 2-level**	<=HS°	91	32.3		20	47.6	χ^2^	0.05
	Any college	101	67.7		22	52.4		
**Anti-inflammatories**	Yes	27	9.5		6	14.3	Fisher’s*	0.41
**Antacids**	Yes	20	7.1		4	9.5	Fisher’s*	0.53
**General health**	Fair/poor	26	9.2		5	11.9	Fisher’s*	0.57
**Treatment (4-level)**	Placebo	87	30.7		9	21.4	χ^2^	0.12
	Antiox‡ only	91	32.2		10	23.8		
	Zinc only	49	17.3		13	31.0		
	Antiox‡+Zn†	56	19.8		10	23.8		
**Treatment (2-level)**	No zinc	178	62.9		19	45.2	χ^2^	0.03
	Yes zinc	105	37.1		23	54.8		
**Treatment (2-level)**	No antiox‡	136	48.1		22	52.4	χ^2^	0.6
	Yes antiox‡	147	51.9		20	47.6		

* Fisher’s exact test,

† Zn: zinc,

‡ Antiox: antioxidant,

° HS: high school,

No: number, SD: standard deviation

**Table 2:** Multivariable modeling: final model including age, education level (any college vs no college), diabetes, and treatment group with same covariates for each treatment variable.

	4-level treatment variable				2-level treatment variable (Zn† vs no Zn†)				2-level treatment variable (Antiox‡ vs no Antiox‡)			
	OR	95% CI		p	OR	95% CI		p	OR	95% CI		p
**Age (per year)**	1.065	0.982	1.155	0.131	1.059	0.978	1.148	0.159	1.072	0.990	1.159	0.085
**Female sex**	–	–	–		–	–	–		–	–	–	
**BMI**	–	–	–		–	–	–		–	–	–	
**Any college**	0.585	0.298	1.147	0.119	0.561	0.288	1.092	0.089	0.560	0.288	1.089	0.088
**Diabetes**	2.271	0.926	5.565	0.073	2.208	0.907	5.377	0.081	2.250	0.931	5.440	0.072
**Ever smoked**	–	–	–		–	–	–		–	–	–	
Tx*: Antiox‡ **only**	1.012	0.386	2.657	0.305	–	–	–		–	–	–	
Tx*: Zn† **only**	2.322	0.906	5.951	0.063	–	–	–		–	–	–	
Tx*: Antiox‡+ **Zn**†	1.488	0.553	4.007	0.782	–	–	–		–	–	–	
**Tx** * **: any Zn** †	–	–	–		1.867	0.958	3.637	0.067	–	–	–	
**Tx** * **: any Antiox** ‡	–	–	–		–	–	–		0.794	0.408	1.545	0.496
**General health (fair/poor)**	–	–	–		–	–	–		–	–	–	
**Anti-inflammatories**	–	–	–		–	–	–		–	–	–	
**Antacids**	–	–	–		–	–	–		–	–	–	

* Tx: treatment group,

† Zn: zinc,

‡ Antiox: antioxidant,

OR: odds ratio, CI: confidence interval

## Appendix A. Supporting information

Supplementary data associated with this article can be found in the online version at doi:10.1016/j.jpain.2025.104790.
