# Supplementary material for: Prevalence and risk factors for the development of chronic postoperative pain after cataract surgery in the Age-related Eye Disease Study (AREDS)
Source: J Pain. Author manuscript; Available in PMC 2026 May 10. (PMC13157973; doi:10.1016/j.jpain.2025.104790)
Supplement: 1 [file NIHMS2171825-supplement-1.docx]

**Supplementary Table 1.** Univariable comparison of those included in the study (cases and controls) and those excluded (people who had cataract surgery but did not have VFQs both before and after surgery)

| **Characteristic** | **Included (n=325)** | | | **Excluded (n=876)** | | | **Test** | **p** |
| --- | --- | --- | --- | --- | --- | --- | --- | --- |
|  | **Mean** | **SD** | **Range** | **Mean** | **SD** | **Range** |  |  |
| **Age (y)** | 69.8 | 4.4 | 58-79 | 70.6 | 4.1 | 62-78 | t-test | 0.003 |
| **BMI** | 27.9 | 5.1 | 18.1-58.4 | 28.8 | 5.4 | 20.8-45.6 | t-test | 0.009 |
|  | **Category** | **No.** | **%** |  | **No.** | **%** |  |  |
| **White race** |  | 316 | 97.2 |  | 847 | 96.7 | χ2 | 0.63 |
| **Female sex** |  | 193 | 59.4 |  | 526 | 60.5 | χ2 | 0.84 |
| **Smoking 3-level** | Never | 146 | 44.9 |  | 387 | 44.2 | χ2 | 0.48 |
|  | Quit | 161 | 49.5 |  | 423 | 48.3 |  |  |
|  | Current | 18 | 5.5 |  | 66 | 7.5 |  |  |
| **Smoking 2-level** | Never | 146 | 44.9 |  | 387 | 44.2 | χ2 | 0.82 |
|  | Ever | 179 | 55.1 |  | 489 | 55.8 |  |  |
| **Diabetes** | Yes | 34 | 10.5 |  | 80 | 9.1 | χ2 | 0.48 |
| **Education 3-level** | <=HS | 111 | 34.3 |  | 322 | 36.8 | χ2 | 0.71 |
|  | Some college | 103 | 31.8 |  | 272 | 31.0 |  |  |
|  | College grad | 110 | 33.9 |  | 282 | 32.2 |  |  |
| **Education 2-level** | <College grad | 214 | 66.0 |  | 594 | 67.8 | χ2 | 0.56 |
|  | College grad | 110 | 34.0 |  | 282 | 32.2 |  |  |
| **Education 2-level** | <=HS | 111 | 34.3 |  | 322 | 36.8 | χ2 | 0.42 |
|  | Any college | 213 | 65.7 |  | 554 | 63.2 |  |  |
| **Anti-inflammatories** | Yes | 33 | 10.2 |  | 110 | 12.6 | χ2 | 0.25 |
| **Antacids** | Yes | 24 | 7.4 |  | 63 | 7.2 | χ2 | 0.91 |
| **General health** | Fair/poor | 31 | 9.5 |  | 73 | 11.5 | χ2 | 0.36 |
| **Treatment (4-level)** | Placebo | 96 | 29.5 |  | 271 | 30.9 | χ2 | 0.79 |
|  | Antiox only | 101 | 31.1 |  | 249 | 28.4 |  |  |
|  | Zinc only | 62 | 19.1 |  | 181 | 20.7 |  |  |
|  | Antiox+Zn | 66 | 20.3 |  | 175 | 20.0 |  |  |
| **Treatment (2-level)** | No zinc | 197 | 60.6 |  | 520 | 59.4 | χ2 | 0.69 |
|  | Yes zinc | 128 | 39.4 |  | 356 | 40.6 |  |  |
| **Treatment (2-level)** | No antiox | 158 | 48.6 |  | 452 | 51.6 | χ2 | 0.32 |
|  | Yes antiox | 167 | 51.4 |  | 424 | 48.4 |  |  |

**Supplementary Table 2.** Multivariable modeling: risk of being a case (changing from no/mild pain before cataract surgery to moderate/severe pain score after cataract surgery) relative to a control (remaining at no/mild pain score after cataract surgery) while adjusting for demographic, medical history, and AREDS treatment group information (age, sex, BMI, diabetes, smoking, level of formal education, self-reported general health (VFQ question), any use of anti-inflammatories, any use of antacids, and treatment group. A full model containing each of the covariates was run separately for each of the three treatment group variables, followed by a reduced model which only included variables with a p-value of <0.10, along with age.

|  | **4-level treatment variable** | | | | | | | | **2-level treatment variable (Zn vs no Zn)** | | | | | | | | **2-level treatment variable (Ax vs no Ax)** | | | | | | | |
| --- | --- | --- | --- | --- | --- | --- | --- | --- | --- | --- | --- | --- | --- | --- | --- | --- | --- | --- | --- | --- | --- | --- | --- | --- |
|  | **Full model** | | | | **Reduced model** | | | | **Full model** | | | | **Reduced model** | | | | **Full model** | | | | **Reduced model** | | | |
|  | **OR** | **95% CI** | | **p** | **OR** | **95% CI** | | **p** | **OR** | **95% CI** | | **p** | **OR** | **95% CI** | | **p** | **OR** | **95% CI** | | **p** | **OR** | **95% CI** | | **p** |
| **Age (per year)** | 1.066 | 0.982 | 1.157 | 0.128 | 1.070 | 0.987 | 1.159 | 0.100 | 1.062 | 0.979 | 1.152 | 0.148 | 1.059 | 0.978 | 1.148 | 0.159 | 1.073 | 0.991 | 1.162 | 0.084 | 1.077 | 0.997 | 1.164 | 1.077 |
| **Female sex** | 0.997 | 0.489 | 2.032 | 0.994 | -- | -- | -- |  | 0.987 | 0.487 | 2.001 | 0.971 | -- | -- | -- |  | 0.992 | 0.489 | 2.010 | 0.982 | -- | -- | -- |  |
| **BMI** | 1.029 | 0.966 | 1.097 | 0.377 | -- | -- | -- |  | 1.029 | 0.966 | 1.095 | 0.379 | -- | -- | -- |  | 1.033 | 0.970 | 1.100 | 0.312 | -- | -- | -- |  |
| **Any college** | 0.579 | 0.290 | 1.159 | 0.123 | -- | -- | -- |  | 0.554 | 0.279 | 1.099 | 0.091 | 0.561 | 0.288 | 1.092 | 0.089 | 0.569 | 0.288 | 1.124 | 0.104 | -- | -- | -- |  |
| **Diabetes** | 2.361 | 0.936 | 5.954 | 0.069 | 2.310 | 0.946 | 5.644 | 0.066 | 2.317 | 0.923 | 5.815 | 0.074 | 2.208 | 0.907 | 5.377 | 0.081 | 2.305 | 0.928 | 5.720 | 0.072 | 2.271 | 0.944 | 5.462 | 2.271 |
| **Ever smoked** | 1.251 | 0.613 | 2.555 | 0.539 | -- | -- | -- |  | 1.268 | 0.623 | 2.582 | 0.513 | -- | -- | -- |  | 1.253 | 0.618 | 2.542 | 0.532 | -- | -- | -- |  |
| **Tx: Antiox only** | 1.031 | 0.389 | 2.733 | 0.337 | 1.039 | 0.397 | 2.717 | 0.315 | -- | -- | -- |  | -- | -- | -- |  | -- | -- | -- |  | -- | -- | -- |  |
| **Tx: Zn only** | 2.267 | 0.874 | 5.881 | 0.085 | 2.496 | 0.981 | 6.349 | 0.038 | -- | -- | -- |  | -- | -- | -- |  | -- | -- | -- |  | -- | -- | -- |  |
| **Tx: Antiox+ Zn** | 1.525 | 0.560 | 4.154 | 0.739 | 1.456 | 0.544 | 3.900 | 0.886 | -- | -- | -- |  | -- | -- | -- |  | -- | -- | -- |  | -- | -- | -- |  |
| **Tx: any Zn** | -- | -- | -- |  | -- | -- | -- |  | 1.849 | 0.937 | 3.649 | 0.076 | 1.867 | 0.958 | 3.637 | 0.067 | -- | -- | -- |  | -- | -- | -- |  |
| **Tx: any Antiox** | -- | -- | -- |  | -- | -- | -- |  | -- | -- | -- |  | -- | -- | -- |  | 0.825 | 0.419 | 1.625 | 0.578 | 0.774 | 0.399 | 1.502 | 0.774 |
| **General health (fair/poor)** | 0.750 | 0.248 | 2.265 | 0.610 | -- | -- | -- |  | 0.713 | 0.237 | 2.145 | 0.547 | -- | -- | -- |  | 0.814 | 0.278 | 2.384 | 0.708 | -- | -- | -- |  |
| **Anti-inflammatories** | 1.534 | 0.571 | 4.126 | 0.396 | -- | -- | -- |  | 1.507 | 0.562 | 4.041 | 0.415 | -- | -- | -- |  | 1.481 | 0.551 | 3.984 | 0.437 | -- | -- | -- |  |
| **Antacids** | 1.126 | 0.337 | 3.769 | 0.847 | -- | -- | -- |  | 1.207 | 0.368 | 3.958 | 0.757 | -- | -- | -- |  | 1.282 | 0.399 | 4.121 | 0.677 | -- | -- | -- |  |

*Tx: treatment group, Zn: zinc, Antiox: antioxidant
