# Supplementary material for: Prevalence and risk factors for the development of chronic postoperative pain after cataract surgery in the Age-related Eye Disease Study (AREDS)
Source: J Pain. Author manuscript; Available in PMC 2026 May 10. (PMC13157973; doi:10.1016/j.jpain.2025.104790)
Supplement: 3 [file NIHMS2171825-supplement-3.docx]

**APPENDIX.**

**AREDS RESEARCH GROUP**

*The Eye Center at Memorial*

Aaron Kassoff, MD, Shalom Kieval, MD, Michel Mehu, JoAnne Buehler, Mary Eglow, RN, and Francine Kaufman.

*Associated Retinal Consultants, PC*

Raymond R. Margherio, MD, Morton S. Cox, MD, Bruce Garretson, MD, Tarek Hassan, MD, Alan Ruby, MD, Michael T. Trese, MD, Jane Camille Werner, MD, George A. Williams, MD, Virginia Regan, RN, Patricia Manatrey, RN, Kristi Cumming, RN, Mary Zajechowski, Rachel Falk, Patricia Streasick, and Lynette Szydlowski.

*Devers Eye Institute*

Richard F. Dreyer, MD, Colin Ma, MD, Carolyn Beardsley, and Harold Crider.

*Emory University*

Antonio Capone Jr, MD, Thomas M. Aaberg, MD, Daniel Martin, MD, David Saperstein, MD, Paul Sternberg, Jr, MD, Linda Curtis, Barbara Stribling, James Gilman, Bob Myles, and Ray Swords.

*Ingalls Memorial Hospital*

David H. Orth, MD, Timothy P. Flood, MD, Joseph Civantos, MD, Serge deBustros, MD, Kirk H. Packo, MD, Celeste MacLeod, Chris Morrison, Douglas A. Bryant, Don Doherty, and Sharon Sandoval.

*Massachusetts Eye and Ear Infirmary*

Johanna M. Seddon, MD, Michael K. Pinnolis, MD, Desiree A. Jones-Devonish, Valerie D. Crouse, MS, Kristin K. Snow, MS, Claudia Evans, OD, Nancy Davis, Charlene Callahan, David Walsh, Jennifer Dubois, and Ilene Burton, RN.

*National Eye Institute Clinical Center*

Frederick L. Ferris, III, MD, Emily Y. Chew, MD, Karl Csaky, MD, PhD, Sally A. McCarthy, RN, MSN, Katherine Hall Dabas, Linda Goodman, Young Ja Kim, RN, BSN, Patrick Lopez, Richard Mercer, Leanne M. Ayres, Toni LaRean, Anne Randall, Marilois Chicca, Patrick F. Ciatto, Ernest Kuehl, Iris Kivitz, and Dessie Koutsandreas.

*University of Pittsburgh*

Thomas R. Friberg, MD, Andrew Eller, MD, Michael B. Gorin, MD, PhD, Jane Alexander, and Barbara Mack.

*The Johns Hopkins Medical Institutions*

Susan B. Bressler, MD, Neil M. Bressler, MD, Gary Cassel, MD, Morton Goldberg, MD, Julia A. Haller, MD, Lois Ratner, MD, Andrew P. Schachat, MD, Steven H. Sherman, MD, Janet S. Sunness, MD, Sherrie Schenning, Catherine Sackett, CANP, Dennis Cain, David Emmert, Terry George, and Stacy Wheeler.

*Elman Retina Group, PA*

Michael J. Elman, MD, Rex Ballinger, OD, Arturo Betancourt, MD, David Glasser, MD, Joyce Lammlein, MD, Ronald Seff, MD, Margin Shuman, MD, JoAnn Starr, Anita Carrigan, Christine Ringrose, Terri Mathews, Peter Sotirakos, and Theresa Cain.

*University of Wisconsin - Madison*

Suresh R. Chandra, MD, Frank L. Myers, MD, T. Michael Nork, MD, Thomas Stevens, MD, Barbara Blodi, MD, Justin Gottlieb, MD, Tracy Perkins, MPH, Margo Blatz, Wendy Walker, Bob Harrison, Gene Knutson, Denise Krolnik, and Guy Somers.

*University of Wisconsin - Reading Center*

Matthew D. Davis, MD, Barbara E.K. Klein, MD, Ronald Klein, MD, Larry Hubbard, MA, Yvonne L. Magli, Judith Brickbauer, Sarah Ansay, Jane Armstrong, Michael Neider, Hugh Wabers, James Baliker, Linda Kastorff, Kristine Laher, Darlene Badal, Patricia L. Geithman, Kathleen D. Miner, William N. King, Kurt R. Osterly, Kristi L. Dohm, James A. Onofrey, Barbara Esser, Cynthia Hurtenback, Marian R. Fisher, Nancy L. Robinson, and James Reimers.

*Centers for Disease Control and Prevention - Central Laboratory*

Dayton Miller, PhD, Barbara Bowman, PhD, Elaine Gunter, and Anne Sowell, PhD.

*Coordinating Center - The EMMES Corporation*

Anne S. Lindblad, PhD, Fred Ederer, MA, FACE, Roy C. Milton, PhD, Gary Gensler, MS, Ravinder Anand, PhD, Gary Entler, Elaine Stine, Stuart H. Berlin, Phyllis R. Scholl, and Susan A. Mengers.

*National Eye Institute Project Office*

Frederick L. Ferris, III, MD, Emily Y. Chew, MD, Robert Sperduto, MD, and Natalie Kurinij, PhD.
